# A Small Volatile Bacterial Molecule Triggers Mitochondrial Dysfunction in Murine Skeletal Muscle

**DOI:** 10.1371/journal.pone.0074528

**Published:** 2013-09-30

**Authors:** A. Aria Tzika, Caterina Constantinou, Arunava Bandyopadhaya, Nikolaos Psychogios, Sangseok Lee, Michael Mindrinos, J. A. Jeevendra Martyn, Ronald G. Tompkins, Laurence G. Rahme

**Affiliations:** 1 Department of Surgery, Harvard Medical School and Massachusetts General Hospital, Boston, Massachusetts, United States of America; 2 Athinoula A. Martinos Center of Biomedical Imaging, Massachusetts General Hospital, Boston, Massachusetts, United States of America; 3 Department of Biochemistry, Stanford University School of Medicine, Stanford, California, United States of America; 4 Department of Anesthesiology and Critical Care, Harvard Medical School and Massachusetts General Hospital, Boston, Massachusetts, United States of America; 5 Shriners Hospitals for Children Boston, Boston, Massachusetts, United States of America; University of North Dakota, United States of America

## Abstract

Mitochondria integrate distinct signals that reflect specific threats to the host, including infection, tissue damage, and metabolic dysfunction; and play a key role in insulin resistance. We have found that the *Pseudomonas aeruginosa* quorum sensing infochemical, 2-amino acetophenone (2-AA), produced during acute and chronic infection in human tissues, including in the lungs of cystic fibrosis (CF) patients, acts as an interkingdom immunomodulatory signal that facilitates pathogen persistence, and host tolerance to infection. Transcriptome results have led to the hypothesis that 2-AA causes further harm to the host by triggering mitochondrial dysfunction in skeletal muscle. As normal skeletal muscle function is essential to survival, and is compromised in many chronic illnesses, including infections and CF-associated muscle wasting, we here determine the global effects of 2-AA on skeletal muscle using high-resolution magic-angle-spinning (HRMAS), proton (^1^H) nuclear magnetic resonance (NMR) metabolomics, *in vivo* ^31^P NMR, whole-genome expression analysis and functional studies. Our results show that 2-AA when injected into mice, induced a biological signature of insulin resistance as determined by ^1^H NMR analysis-, and dramatically altered insulin signaling, glucose transport, and mitochondrial function. Genes including Glut4, IRS1, PPAR-γ, PGC1 and Sirt1 were downregulated, whereas uncoupling protein UCP3 was up-regulated, in accordance with mitochondrial dysfunction. Although 2-AA did not alter high-energy phosphates or pH by *in vivo* ^31^P NMR analysis, it significantly reduced the rate of ATP synthesis. This affect was corroborated by results demonstrating down-regulation of the expression of genes involved in energy production and muscle function, and was further validated by muscle function studies. Together, these results further demonstrate that 2-AA, acts as a mediator of interkingdom modulation, and likely effects insulin resistance associated with a molecular signature of mitochondrial dysfunction in skeletal muscle. Reduced energy production and mitochondrial dysfunctional may further favor infection, and be an important step in the establishment of chronic and persistent infections.

## Introduction

Pathogens modulate host cell functions to promote their own survival within the host dynamic environment, and to evade the host immune system by hijacking functions of cell organelles, including plasma rafts [1], golgi [2], and mitochondria [3]. Mitochondria act as signaling platforms in diverse biological processes, including apotosis [3], metabolism, and innate immune signaling [4]. Bacterial small molecules can alter mitochondrial function [5], [6], with bacterial effector proteins and toxins primarily affecting mitochondrial programmed apotosis [3]; and bacterial membrane constituents, such as lipopolysaccharide (LPS) and peptidoglycan (PGN) triggering metabolic diseases, including insulin resistance [7].

Environmental stimuli can alter mitochondrial function via coordinated changes in gene expression [8]. For instance, specific members of the peroxisomal proliferator activator receptor (PPAR)-γ and proliferator activator receptor (PPAR)-γ coactivator (PGC1) gene families respond to physiological stimuli to regulate genes that control mitochondrial biogenesis [9], nuclear and mitochondrial oxidative metabolism, tricarboxylic acid (TCA) cycle enzymes, whole body glucose homeostasis, and lipid oxidation and electron transport complexes [10]. Also, cellular stress and infection are sensed by the innate immune system by pattern recognition receptors, which upon activation, initiate defense and repair pathways [11], [12]. It is possible that, similarly to viruses, bacteria may activate the inflammasome via altered cell metabolism and mitochondrial activity [13]–[16].

Metabolic responses begin promptly upon the initiation of infection, and progress as a series of coordinated events [17]. Mitochondria may play a key role in the development of insulin resistance [18]. For example, glucose stimulated insulin secretion from pancreatic β cells requires intact mitochondrial function [19]: modifications in mitochondrial oxidative activity and mitochondrial adenosine triphosphate synthesis is linked to insulin resistance [19], and these changes involve the up-regulation of mitochondrial uncoupling proteins (UCPs) [20]; altered mitochondrial fatty acid oxidation, together with the accumulation of intracellular fatty acid metabolites (acyl-CoA and diacylglycerol), disrupts insulin signaling, a phenomenon exacerbated by free fatty acids (FFA) [21]; and genes involved in mitochondria biogenesis such as the PPAR-γ and PGC-1 (α and β are down-regulated in patients with insulin resistance [22], [23] and possibly also down-regulated in insulin resistant cystic fibrosis (CF) patients [24], [25].

CF patients are particularly susceptible to highly problematic *Pseudomonas aeruginosa* infections. This pathogen, which causes chronic infections that are often intractable to traditional antibiotic therapy [26],[27], employs cell-to-cell communication systems, termed quorum sensing (QS). QS regulates collective behaviors, including virulence, that depend on the actions of specific excreted diffusible small molecular signals, termed infochemicals [28], [29]. QS infochemicals also act as immunomodulatory signals [30], [31], and respiratory chain inhibitors [6]. The infochemical 2-amino-acetophenon (2-AA) [32], [33] signals phenotypic changes in the pathogen [34], and modulates host immune responses [31] that favor chronic infections, and potentially compromise host metabolism.

Here we employ metabolomics, genomics, and functional analyses to interrogate the *in vivo* 2-AA effects on mitochondrial function. We use Nuclear Magnetic Resonance (NMR) spectroscopy, which can demonstrate mitochondrial dysfunction [35],[36] to assess physiological and metabolic biomarkers in intact muscle; and *in vivo* NMR, to assess functional mitochondrial metabolism. This technique is superior to biopsy-based genomic analysis, which can only interrogate mitochondrial capacity versus function [37]. Our results show that 2-AA, beyond its previously identified immunomodulatory activity [31], triggers host metabolic changes that occur concurrently with mitochondrial and skeletal muscle dysfunction, to promote pathogenicity.

## Materials and Methods

### Experimental animals

6-wk-old male CD1 mice weighing approximately 20–25 g were purchased from Charles River Laboratory (Boston, MA). The animals were maintained on a regular light-dark cycle (lights on from 8∶00 h to 20∶00 h) at an ambient temperature of 22±1°C, with free access to food and water. Mice were injected intra-peritoneally (IP) with 100 µl of 2-AA (6.75 mg /kg mice), and mouse skeletal muscle was analyzed 4 days post 2-AA treatment. *In vivo* ^31^P NMR spectroscopy was performed on intact mice, and *ex- vivo* ^1^H NMR spectroscopy was performed on intact gastrocnemious muscle samples.

### Ethics Statement

This study was carried out in strict accordance with the recommendations of the Guide for the Care and Use of Laboratory Animals of the National Institutes of Health. The protocol was approved by the Committee on the Ethics of Animal Experiments at Massachusetts General Hospital (Permit Number: 2006N000093/2). All procedures were performed under sodium pentobarbital anesthesia, and every effort was made to minimize suffering.

### ^31^P NMR spectroscopy

#### Data acquisition

The theoretical basis of saturation transfer experiments and calculations were as described by Forsen and Hoffman [36], [38]. Animals were analyzed using *in vivo* ^31^P NMR spectroscopy 4 days post 2-AA treatment. Mice were transiently anesthetized with isoflurane (3.0%) plus O_2_ (2.0 l/min) delivered through a nose cone, and then placed in a customized restraining tube. Each animal's right hind limb was placed into a solenoid coil (four turns; length, 2 cm; diameter, 1 cm) tuned to ^31^P frequency (162.1 MHz). During MR imaging, mice were continuously anesthetized with isoflurane (1.5%) plus O_2_ (1.0 l/min). The rectal body temperature was maintained at 37±1°C using heated water blankets. All *in vivo* ^31^P NMR experiments were performed in a horizontal bore magnet (proton frequency at 400 MHz, 21 cm diameter, Magnex Scientific, Varian, Palo Alto, CA, USA) using a Bruker Advance console. Field homogeneity was adjusted using the ^1^H signal of tissue water. A 90° pulse was optimized for detection of phosphorus spectra (repetition time 2 s, 400 averages, 4,096 data points). Saturation 90° selective pulse trains (duration, 36.533 ms; bandwidth, 75 Hz) followed by crushing gradients were used to saturate the γ-ATP peak. The same saturation pulse train was also applied downfield of the inorganic phosphate (Pi) resonance, symmetrically to the γ-ATP resonance. T1 relaxation times of Pi and phosphocreatine (PCr) were measured using an inversion recovery pulse sequence in the presence of γ-ATP saturation. An adiabatic pulse (400 scans; sweep width, 10 kHz; 4,000 data points) was used to invert Pi and PCr, with an inversion time between 152 and 7,651 ms.

#### Data analysis

^31^P NMR spectra were analyzed using the MestReNova NMR software package (Mestrelab Research S.L., v. 6.2.1 NMR solutions, Website: www.mestrec.com). Free induction decays were zero-filled to 8,192 points and apodized with exponential multiplication (30 Hz) before Fourier transformation. The spectra were then manually phased and corrected for baseline broad features using the Whittaker smoother algorithm [39]. The Levenberg–Marquardt algorithm was used to least-square-fit a model of mixed Gaussian/Lorentzian functions to the data. Similarly, the *T*1_obs_ relaxation time for Pi and PCr was calculated by fitting the function *y*  =  *A*1 (1– *A*2*e* ^−(*t/T*1obs)^) to the inversion recovery data, where *y* is the *z* magnetization, and *t* is the inversion time.

#### Calculation of intramyocellular pH

The formula pH  = 6.75+ log[(s–3.27)/(5.69–s)], where s is the chemical shift difference (in ppm) between the Pi and the PCr peaks [40] was used to calculate intramyocallular pH.

#### Calculation of ATP concentration

ATP concentration was measured using the Bioluminescence Assay Kit CLS II, Cat# 1699695 (Roche Diagnostics Corporation, Indianapolis, IN 46250–0414, USA).

#### Calculation of ATP synthesis rate

^31^P-NMR spectra data, and the ATP concentration, were used to calculate the ATP synthesis rate, as described by Forsen and Hoffman [38]. In brief, the chemical reaction between Pi and ATP is:
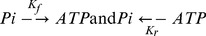
(1)Where *k_f_* and *k_r_* are reaction rate constants in each direction. The influence of the chemical exchange between Pi and ATP on the longitudinal magnetization 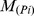
of Pi is described by:



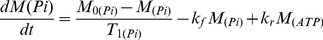
(2)At equilibrium 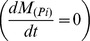 so at saturated ATP, 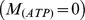 the equation (2) becomes
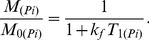
(3)


The spin lattice relaxation time T_1app_, measured using the inversion recovery pulse sequence in the presence of the ATP saturation, is related to the intrinsic T_1(Pi)_ by:
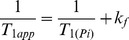
(4)


Combining (3) and (4) gives:
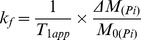
(5)where 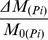
is the fractional change of the longitudinal magnetization 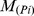
of Pi. All the quantities on the right side of (5) can be calculated from the NMR data. Finally the unidirectional ATP synthesis flux can be calculated as
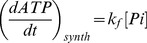
(6)where Pi is the concentration of Pi extrapolated from the baseline NMR spectrum by comparing the peak integrals from Pi and γ-ATP, with respect to ATP concentration.

### Extraction of RNA Samples

The left gastrocnemius muscle was harvested at 4 days post 2-AA treatment (n = 3, for each time point), to determine changes in whole muscle gene expression. Mice were anesthetized by IP injection of 40 mg/kg pentobarbital, and the muscle specimens were excised and immediately immersed in 1 ml Trizol (GibcoBRL, Invitrogen, Carlsbad, CA) for RNA extraction. All mice were then administered a lethal dose of pentobarbital (200 mg/kg) by IP injection. Each muscle specimen was homogenized for 60 s with a Brinkman Polytron 3000 before total RNA extraction. Chloroform (200 μl) was added to the homogenized muscle and mixed by inverting the tube for 15 s. After centrifugation at 12000×*g* for 15 min, the upper aqueous phase was collected and precipitated by adding 500 μl isopropanol. Further centrifugation at 12000×*g* for 10 min separated the RNA pellet, which was then washed with 500 μl of 70% ethanol and centrifuged at 7500×*g* for 5 min prior to air drying. The pellet was resuspended in 100 μl DEPC-H20. An RNeasy Kit (Qiagen, Maryland, USA) was used to purify the RNA according to the manufacturer's protocol. Purified RNA was quantified by UV absorbance at 260 nm and 280 nm, and stored at −70°C for DNA microarray analysis.

### High-Resolution Magic Angle Spinning (HRMAS) ^1^H NMR spectroscopy of intact skeletal muscle tissue

At 4 days post 2-AA treatment, three experimental and three untreated control animals were analyzed with HRMAS ^1^H NMR. The skeletal muscle tissue underlying the hind limb burn site was harvested, immediately frozen in liquid nitrogen, and stored at −80°C. HRMAS ^1^H NMR spectroscopy of muscle tissue was performed on a Bruker Bio-Spin Avance NMR spectrometer (proton frequency at 600.13 MHz, 89 mm Vertical Bore) using a 4-mm triple resonance (^1^H, ^13^C, ^2^H) HRMAS probe (Bruker, Billerica, Massachusetts). The temperature was maintained at 4°C with a BTO-2000 thermocouple unit in combination with a magic angle spinning (MAS) pneumatic unit (Bruker). The MAS speed was stabilized at 4.0±0.001 kHz with a MAS speed controller. ^1^H NMR spectra were acquired using a Carr-Purcell-Meiboom-Gill (CPMG) spin echo pulse sequence, (90°-(τ-180°-τ)_n_-acquisition), with an inter-pulse delay (τ) of 250 µs. Hard 90° (8 µs) and 180° (16 µs) pulse trains were employed. The relaxation delay was set to 2 s, and spectra were collected both with and without water suppression. The transverse relaxation time (T_2_) was measured using the same CPMG pulse sequence by varying n from 0 to 520. Free induction decay (FID) signals were acquired with 8 k points, 600 ms acquisition time, 8 dummy scans, and 128 scans.

HRMAS ^1^H NMR spectra were analyzed using the MestRe-C NMR software package (Mestrelab Research, Santiago de Compostela, Spain, www.mestrec.com). FIDs were zero-filled to 16 k points, and apodized with exponential multiplication (1 Hz) before Fourier transformation. The spectra were then manually phased and corrected for baseline broadening (Whittaker smoother, smooth factor 10,000). The Levenberg-Marquardt algorithm was used to least-squares-fit a model of mixed Gaussian/Lorentzian functions to the data.

The (CH_2_)_n−2_ peak at 1.32 ppm was selected for quantification of intramyocellular lipids (IMCL). Because the sample was spun at magic angle, and the sample volume was much smaller (25 µl) and more homogeneous (reduced bulk magnetic susceptibility effects) than the typical voxel size (1 ml) of *in vivo* ^1^H MRS, no chemical shift difference was observed between IMCL and extramyocellular lipids (EMCL). The small size of the muscle biopsies, and the fact that the samples were collected from the most myocellular part of the muscle, suggest that the main contribution to the (CH_2_) _n−2_ peak was from IMCL lipids.

### Absolute quantification of metabolites from 1-D CPMG spectra

Resonance intensities were measured for -CH_3_ protons of trimethylsilyl- propionic-2,2,3,3-d4 acid (TSP), and compared to the resonance intensities measured for metabolite. The peak intensities of most of the metabolites, as well as of TSP, were calculated from the intensity of the respective resonance (X) measured from the T2-filtered HRMAS ^1^H MR spectrum. The calculated peak intensities were then corrected for T2 relaxation, using Ic(X)  =  Ir(X) * exp(T_CPMG_/T2(X))/n, where Ir(X) is the measured intensity, T_CPMG_ is the CPMG echo time, and n is the number of protons in the functional group, and corresponds to the resonance of the metabolite. In accordance with the “external standard” technique [41], metabolite concentrations were quantified relative to the absolute concentration (μmol) of the respective metabolite (M)  =  Ic(M)/(Ic_TSP_(M) * wt), where wt is the weight of the sample in grams.

### Statistical analysis of HRMAS ^1^H NMR spectroscopy data

Data are reported as means ± standard errors of the mean. Between-groups comparison was performed using analysis of variance with Bonferroni correction for multiple comparisons. Criterion for significance was p<0.0125 corrected. Comparison between measurements was performed in each group with t-test (two-tailed, p<0.0125). All analysis was performed using SPSS (SPSS v12, SPSS Inc.).

### Microarray hybridization

Biotinylated cRNA was generated with 10 µg of total cellular RNA according to the protocol outlined by Affymetrix Inc. (Santa Clara, CA, USA). The cRNA was hybridized onto MOE430A oligonucleotide arrays (Affymetrix, CA, USA), stained, washed, and scanned according to the Affymetrix protocol.

### Genomic data analysis

Data files of the scanned image files hybridized with probes from RNA extracted from the gastrocnemius muscle isolated at the specified times from experimental and control mice (n = 3) were converted to cell intensity files (.CEL files) with the Microarray suite 5.0 (MAS, Affymetrix, CA, USA). The data were scaled to a target intensity of 500, and all possible pairwise array comparisons of the replicates to normal control mice were performed for each time point (i.e., four combinations when two arrays from each time point were compared to the two arrays hybridized to RNA from control mice) using a MAS 5.0 change call algorithm. Probe sets that had a signal value difference greater than 100 and for which one of the two samples being compared was not called “absent”, were scored as differentially modulated when 1) the number of change calls in the same direction were at least 3, 4, and 6, when the number of comparisons were 4, 6, and 9, respectively; and 2) the other comparisons were unchanged. Based on the ratios of 100 genes determined to be invariant in most conditions tested (Affymetrix, CA), an additional constraint of a minimum ratio of 1.65 was applied to control for the known false positives at 5%. The Microarray data is available in http://www.ncbi.nlm.nih.gov/geo/info/linking.html and the accession number is GSE43779. GO analysis has been performed using the GeneSpring GX software (version 11) by Agilent Technologies.

### Cell culture

The C2C12 murine skeletal muscle cell line (American type culture collection, Bethesda, MD) was maintained in Dulbecco's modified Eagle's medium (DMEM, Gibco) supplemented with 10% fetal bovine serum (Gibco) containing penicillin/streptomycin and gentamycin (Gibco) in the presence of 5% CO_2_ at 37°C. The cells were seeded in T-75 tissue culture flasks (Falcon, USA) and used between passages 2 and 3.

### 2-AA cell treatment

C2C12 cells were plated at a density of 10^5^/ml in 6-well plates and grown overnight at 37°C in 5% CO_2_. Cells in the treatment groups were treated with 0.8 mM 2-AA for 24, 36 and 48 h, and then 2-AA treated or non-treated cells were washed with PBS.

### Western blot analysis

Cellular extracts were prepared in RIPA buffer (Cell Signaling Technology). Twenty five micrograms of total protein were added to Laemmli buffer, boiled for 5 min, resolved by 7.5% or 10% polyacrylamide gel electrophoresis (PAGE) in Tris/glycine/SDS buffer (25 mM Tris, 250 mM glycine, 0.1% SDS), and transferred to PVDF membranes (Bio-Rad, Hercules, CA). The membranes were blocked for 2 h in TBS-T (20 mM Tris-HCL, 150 mM NaCl, 0.1% Tween20) containing 5% non-fat milk. The membranes were then washed three times in TBS-T and probed overnight with rabbit antibodies specific for goat PPAR-γ, rabbit SIRT-1, mouse monoclonal PGC-1β (Santa Cruz Biotechnology, Inc., Santa Cruz, CA) at a dilution of 1∶1,000, and mouse α-tubulin (Santa Cruz Biotechnology, Inc) at a dilution of 1∶2,000. Following three washes in TBS-T, the membranes were incubated with secondary horse-radish peroxidase (HRP)-conjugated rabbit anti goat IgG, goat anti-rabbit IgG (Santa Cruz Biotechnology, Inc) or goat anti-mouse IgG secondary antibodies (Promega, Madison, WI), respectively for 1 h, and then washed three times in TBS-T. The blots were visualized with SuperSignal West Pico Chemiluminescent Substrate (Thermo Scientific, Rockford, IL), according to the manufacturer's instructions.

### MTT assay for cell cytotoxicity

The cytotoxicity of cells treated with 0.8 mM 2-AA was measured by MTT assay as previously described [31].

### Plasma FFA assay

Blood was drawn by heart puncture at the time of sacrifice, and the plasma FFA level was measured via the calorimetric FFA assay kit that uses acylation of coenzyme A (NEFA C; Wako Chemicals USA, Inc., Richmond, VA).

### Neuromuscular Function Studies

Functional neuromuscular studies were performed to determine the tensions developed after perturbation with the bacterial molecule and test the integrity of neurotransmission at 4 days post 2-AA treatment. Mice were anesthetized with pentobarbital (60–70 mg/kg, IP), with adequate depth of anesthesia confirmed by the absence of the withdrawal response to toe clamping. Anesthesia was maintained with supplemental intermittent doses of pentobarbital (10–20 mg/kg, IP), every 15–20 minutes. The body temperature was monitored using a rectal thermistor and maintained at 35.5–37°C with a heat lamp. Neuromuscular transmission was monitored by evoked mechanomyography using a peripheral nerve stimulator (NS252, Fisher & Paykel Health Care, Irvine, CA) along with a Grass Force transducer and corresponding software (Grass Instruments, Quincy, MA). With the mice in dorsal recumbency, the tendon of insertion of the tibialis muscle was surgically exposed on each side, and individually attached to separate grass FT03 force displacement transducers. The sciatic nerve was exposed at its exit from the lumbosacral plexus at the thigh and tied with ligatures for indirect nerve stimulation of the muscles. Distal to the ligatures, stimulation electrodes were attached for nerve-mediated stimulation of the tibialis muscle. The knee was rigidly stabilized with a clamp to prevent limb movement during nerve stimulation. Baseline tensions of 10 grams, which yielded optimal evoked tensions, were applied on the immobilized and sham-immobilized tibialis muscles. The sciatic-nerve-evoked tensions of the respective tibialis muscles that were calibrated in grams of force were recorded via a Grass P122 amplifier and displayed using the Grass Polyview Software (Grass Instruments, Quincy, MA). Supramaximal electrical stimuli of 0.2 msec duration were applied to the sciatic nerve at 2 Hz for 2 sec (train-of-four pattern, TOF), every 20 sec, using a Grass S88 stimulator and SIU5 stimulus isolation unit (Grass Instruments, Quincy, MA). The evoked muscle tension developed during TOF stimulation was recorded at the end of 15-min. This was followed by tetanic stimulation at 50 Hz for 5 s to assess the maximal tetanic muscle tension, and the muscle fade associated with stimulus. All values are expressed as mean ± S.E.M. (standard errors of mean). Differences between the two sides of the 2-AA group and control mice were compared using two-way ANOVA test and Bonferroni's multiple comparison tests as a *Post hoc* testing (GraphPad Prism version 5.00 for Windows, GraphPad Software, San Diego California USA, www.graphpad.com). Differences of p value <0.05 were designated as significant.

## Results

### 2-AA reduces ATP synthesis rate and energy production gene expression in skeletal muscle without significantly altering high-energy phosphates or pH

*In vivo* and *ex vivo* NMR magnetic spectroscopy allows measurements of physiological and metabolic biomarkers in intact systems [35], [36]. We used *in vivo* ^31^P NMR to assess the rate of ATP synthesis, and whether 2-AA alters muscle concentrations of high-energy phosphates as a function of perturbed mitochondrial function.

The levels of phosphomonoesters and inorganic phosphate and the ratio Pi/PCr were decreased in the 2-AA treated mice versus the control mice, whereas the levels of PCr were somewhat higher in the 2-AA versus the controls, but not significantly. The intramyocellular pH was not significantly different in the experimental versus control animals (7.26±0.06 and 7.30±0.05, respectively (p = 0.60)).

**Figure 1:**
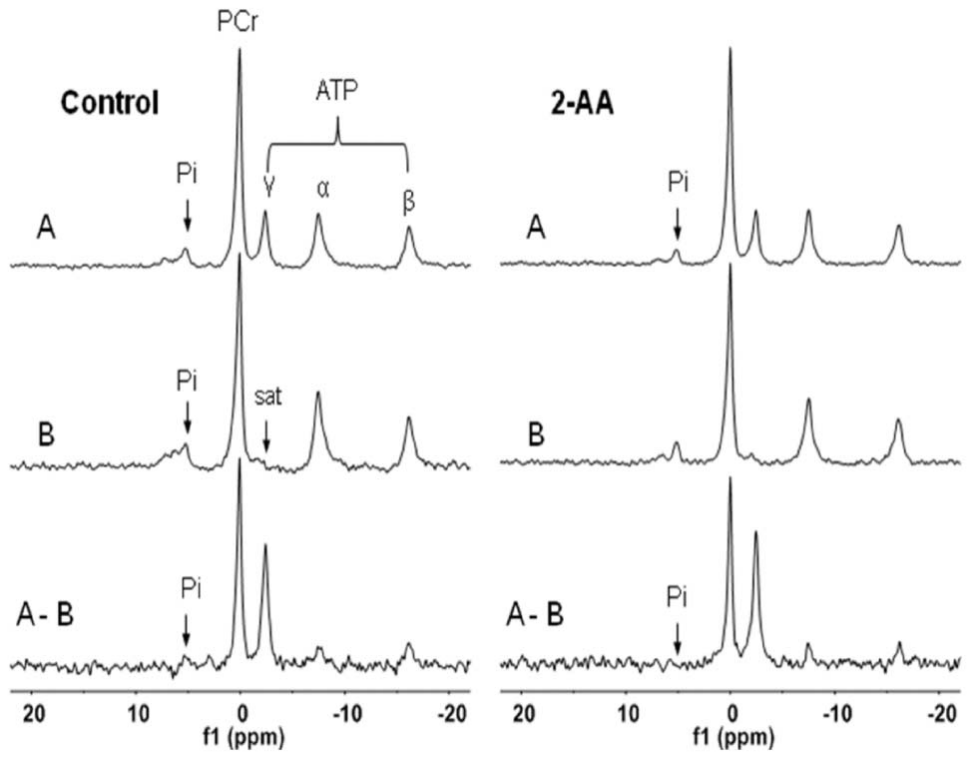
NMR spectra of *in vivo* ^31^P NMR saturation-transfer on mouse hind limb skeletal muscle. Representative summed ^31^P-NMR spectra acquired from control and 2-AA treated mice at day 4, before (A) and after (B) saturation of the γ-ATP resonance, with the difference spectrum between the two shown below (A–B). The arrow on γ-ATP indicates the position of saturation (sat) by rf irradiation (−2.4 ppm). ppm, chemical shift in parts per million.

^31^P NMR spectra were acquired from control and 2-AA treated mice at day 4, before and after saturation of the γ-ATP resonance. Figure 1 shows the summed spectra (non-saturated (A)), upon saturation of the γ-ATP peak (B) with the difference spectrum (A-B)) from the 6 control and the 6 2-AA-treated mice, with the mean results presented in Table 1. The synthesis rates were derived from the NMR data and the ATP concentration assays, with both analyses demonstrating significantly decreased ATP levels in the 2-AA versus the control mice. The fractional change, Δ*M/M_0_*, and the observed spin lattice relaxation time, T_1app_, were used to calculate the *K_f_* rate constant using the equation 
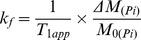
 for the Pi → γ-ATP reaction or 
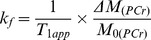
 for the PCr → γ-ATP reaction. The ATP synthesis rate was obtained as the product of *K_f_* and P_i_ concentration. The NMR-measured fractional change for the P_i_ → γ-ATP reaction Δ*M/M_0_* decreased by 30.6% in the 2-AA mice versus the controls (percent change in Δ*M/M_0_*, Table 1). The ATP concentration was lower in 2-AA mice versus controls by approximately 81% (p<0.001 in the unidirectional (one-tailed) t-test (Table 1). Likewise, the ATP synthesis rate was reduced by 90% in the 2-AA versus control mice (p<0.001unidirectional (one-tailed) t-test). Accordingly, the unidirectional ATP synthesis rate for the PCr → γ-ATP reaction was significantly decreased by 82% in the 2-AA versus control mice (one-tailed t-test, p<0.001).

**Table 1:** *In vivo* ^31^P-NMR saturation transfer analysis of limb skeletal muscle from control and 2-AA treated mice.

	Control (*n* = 6)	2-AA (*n* = 6)	Δ (%)	p-value	
***ATP synthesis flux (reaction Pi→*** **γ-** ***ATP)***					
Δ*M/M_0_*	0.33±0.04	0.23±0.02	−30.6		0.015
T_1obs_ (s)	1.13±0.24	1.26±0.19	11.5		NS
*Κ_f_* (s^−1^)	0.29±0.07	0.18±0.03	−37.9		0.005
ATP (μmol/g)	1.19±0.28	0.22±0.05	−81.3		<0.001
P_i_ (μmol/g)	0.39±0.13	0.06±0.02	−84.2		0.011
ATP synthesis rate (μmol/g/s)	0.113±0.047	0.011±0.004	−90.3		0.008
***ATP synthesis flux (reaction PCr→*** **γ-** ***ATP)***					
Δ*M/M_0_*	0.34±0.01	0.31±0.02	−8.1	(NS)	
T_1obs_ (s)	1.59±0.20	1.65±0.21	3.8	(NS)	
*Κ_f_* (s^−1^)	0.21±0.03	0.19±0.03	−11.4	(NS)	
ATP (μmol/g)	1.19±0.28	0.22±0.05	−81.3	<0.001	
PCr (μmol/g)	4.19±1.14	0.85±0.24	−79.7	<0.001	
ATP synthesis rate (μmol/g/s)	0.884±0.267	0.159±0.049	−82.0	<0.001	

Values are means ± SEM; ΔM/M_0_ is the fractional change in P_i_ or PCr magnetization as a result of saturation transfer; T_1obs_ is the observed spin lattice relaxation time of P_i_ or PCr during γ-ATP saturation in seconds; *Κ_f_* is the rate constant for the reactions P_i_ → γ-ATP and PCr → γ-ATP, calculated as (1/T_1obs_) × (Δ*M/M_0_*). ATP synthesis is calculated as P_i_ or PCr × *Κ_f_*. NS: not significant. Unpaired one-tailed Student's t-test was used for the comparisons.

**Figure 2:**
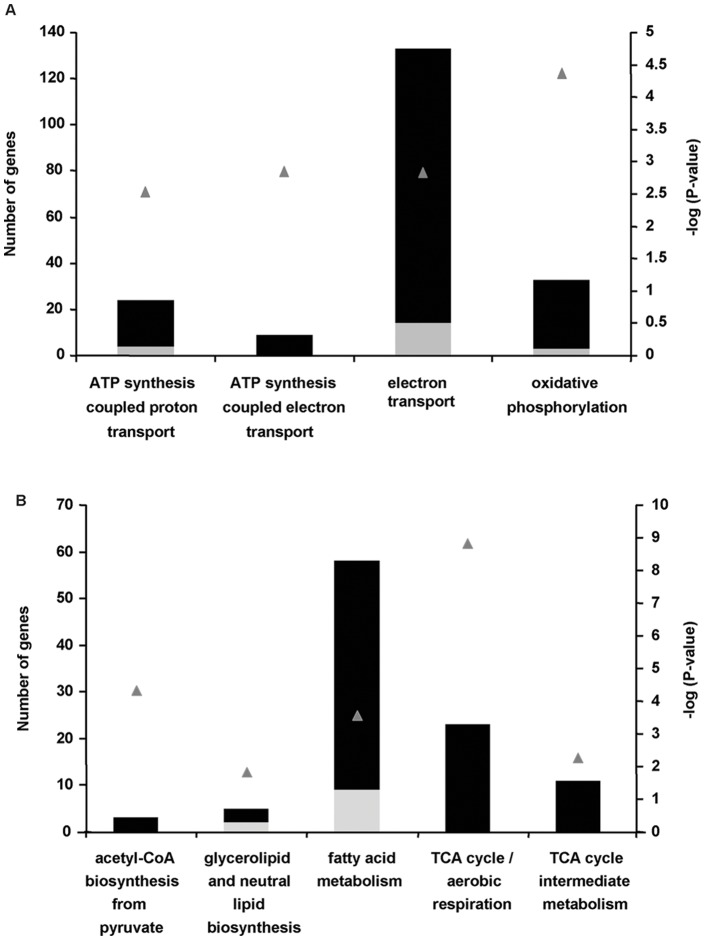
2-AA treatment differentially modulates the genes involved in energy production and intermediate metabolism in skeletal muscle. Differentially expressed genes involved in energy production (A) and intermediate metabolism (B) in response to 2-AA treatment. Grey boxes represent up–egulation, and black boxes represent down-regulation, of the respective gene in muscle from 2-AA treated versus control mice. The negative log10 of p-values represented by gray triangles are shown in the right vertical axis. The expression of certain key genes is down-regulated, consistent with the *in vivo* ^31^P-NMR data (Figure 1 and Table 2).

Figure 2 presents the transcriptome results for the expression of energy production (Fig. 2A) and intermediate metabolism (Fig. 2B) genes, and demonstrates their expression was lower in the skeletal muscle of the 2-AA versus control mice. Several components of the mitochondrial respiratory (proton transport and/or electron transport) chain were down-regulated, including subunits of NADH dehydrogenases and ATP synthase (F_1_F_0_ ATPase or complex V). Also genes involved in oxidative phosphorylation and genes for acetyl-coA biosynthesis from pyruvate (Fig. 2B) were significantly down-regulated. Moreover, the downstream TCA cycle genes, including pyruvate dehydrogenase (lipoamide) beta, succinate dehydrogenase subunits, and citrate synthase were down-regulated. These results strongly suggest that 2-AA leads to metabolic dysfunction in skeletal muscle.

### 2-AA increases insulin resistance biomarker IMCLs and reprograms the expression of key metabolic genes

**Figure 3:**
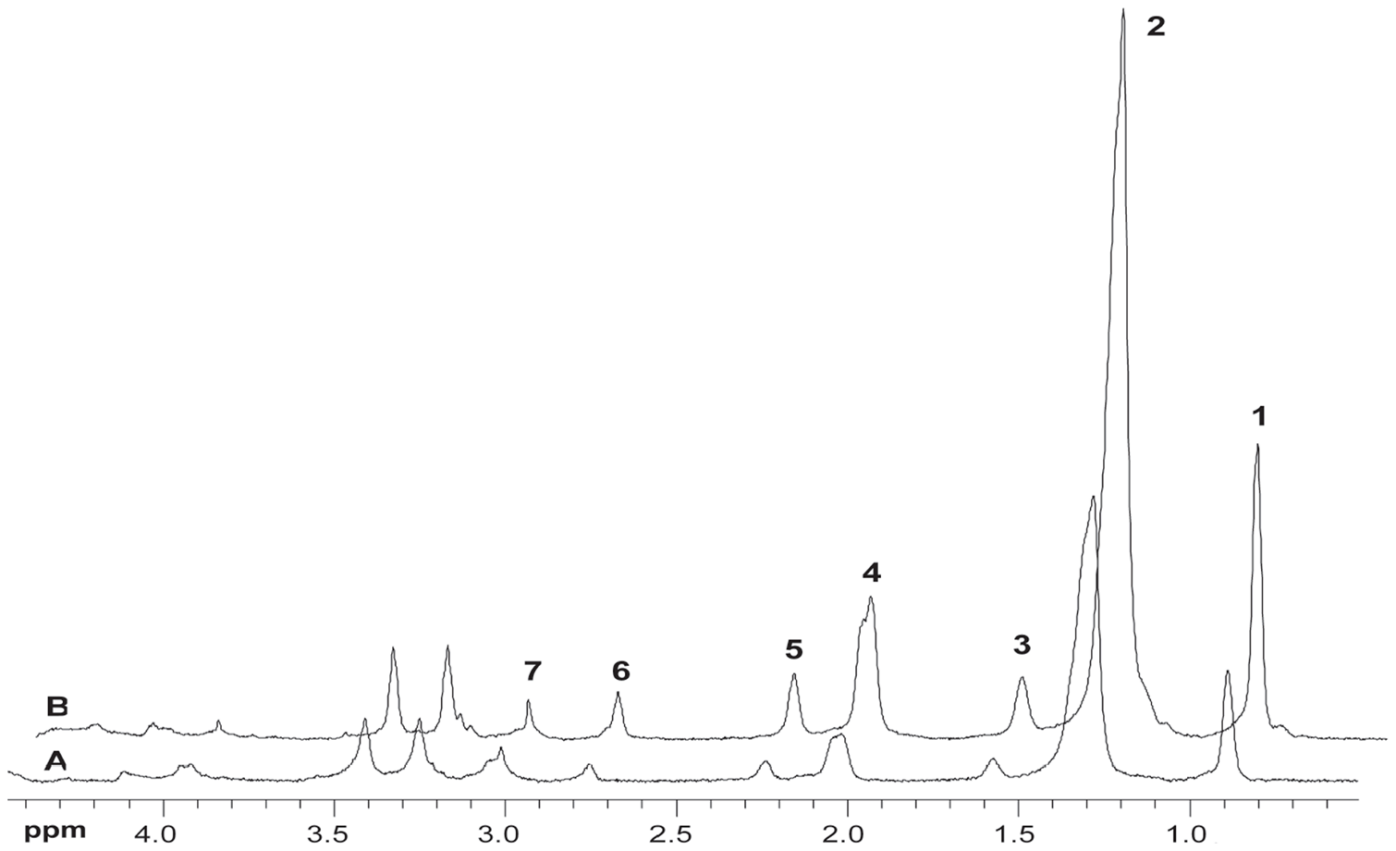
NMR spectra from ^1^H-NMR HRMAS analysis of gastrocnemius skeletal muscle from 2-AA treated versus control mice. The spectra were acquired from 2-AA treated mice at 4 d versus control mice, and scaled to the phosphocreatine plus creatine peak (3.02 ppm). Resonance signals of lipids correspond to: 1) terminal methyl CH_3_ protons (0.9 ppm); 2) acyl chain methylene protons (CH_2_)_n_ of intramyocellular lipids (IMCLs) (1.3 ppm); 3) methylene protons CH_2_C-CO (1.6 ppm); 4) allylic methylene protons C = C-CH_2_-C of monounsaturated fatty acyl moieties (MUFAs) (2.05 ppm); 5) α methylene protons CH_2_CO (2.25 ppm); 6) diallylic methylene protons  = C-CH_2_-C =  of polyunsaturated fatty acyl moieties (PUFAs); and 7) N-methyl protons of phosphocreatine and creatine (3.0 ppm), respectively. The NMR spectra demonstrate increased biomarkers of insulin resistance IMCLs.

**Figure 4:**
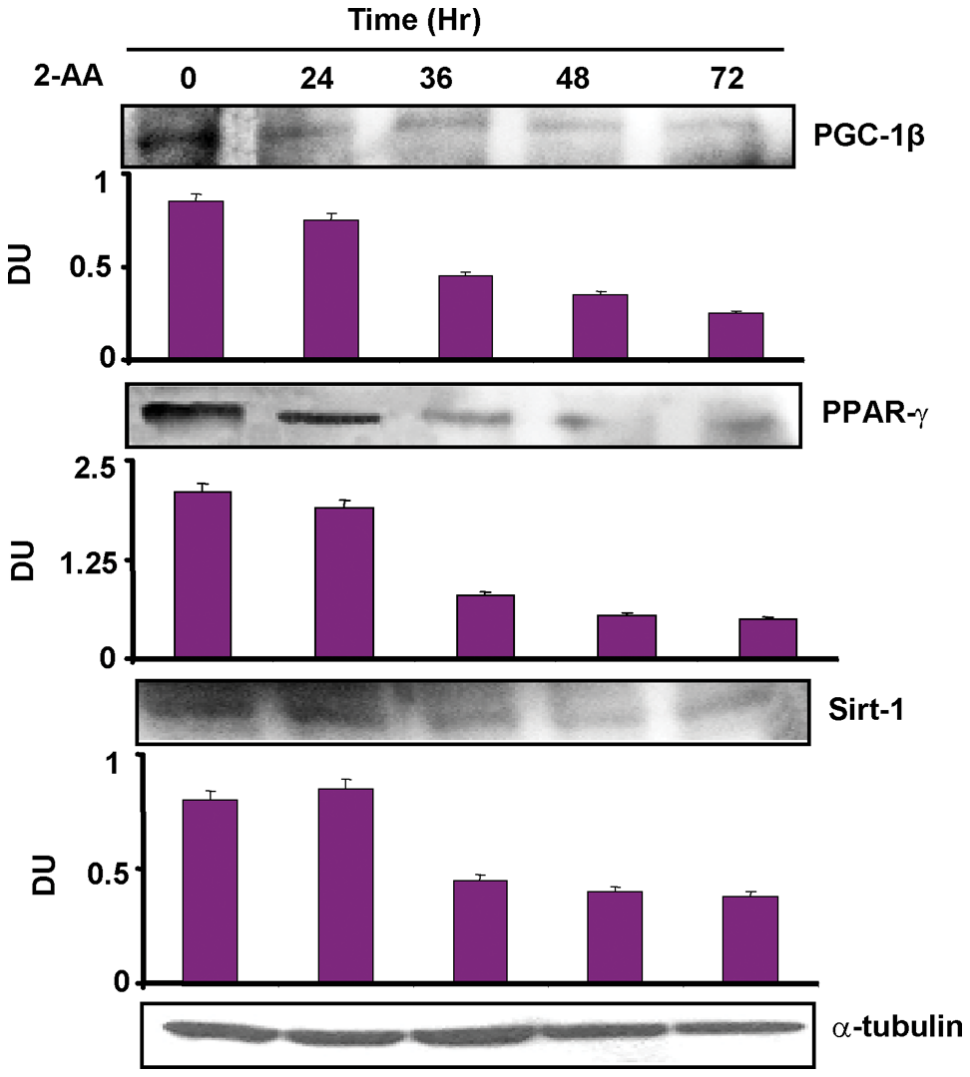
2-AA treatment dampens key metabolic protein levels in mouse skeletal muscle cells. Western blotting of cellular extracts with specific antibodies of PGC-1β, PPAR-γ and Sirt-1 in 2-AA treated cells at the indicated time points. One representative experiment (out of three) is shown. Loading was normalized relative to mouse α-tubulin. Densitometric data are the average of three replicate experiments and are expressed as mean ± SD (vertical bars).*p<0.05 vs. naïve. DU, densitometric units.

**Figure 5:**
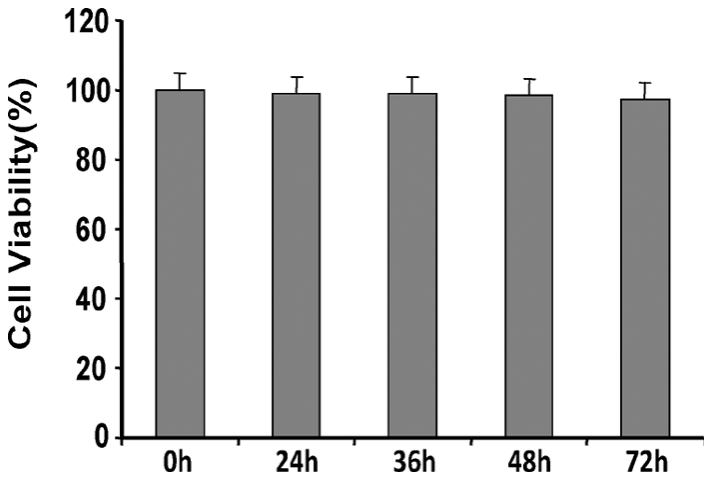
Effects of 2-AA on viability of mouse muscle cells. MTT assay measuring cell viability in mouse skeletal muscle cells (C2C12) after treatment with 0.8 mM 2-AA over time, as indicated in the figure. SDs (vertical bars) were calculated from three replicate experiments.

IMCL levels [42], [43] can serve as useful indices of insulin resistance/metabolic abnormalities in non-vertebrates [44], and vertebrates [45]–[47], including obese and/or type 2 diabetic patients, where increased IMCL levels are due to impaired insulin-stimulated glucose uptake [37], [48], [49]. Figure 3 shows representative ^1^H-NMR spectra from control and 2-AA mice. These results demonstrate a notable rise in IMCLs after 2-AA treatment. Quantitative results of these data (Table 2) demonstrate a significant rise in ICML 4 days post 2-AA treatment. Correspondingly, the expression of genes involved in insulin signaling (i.e., IRS1) and glucose transport (i.e., GLUT4) were down-regulated (Table 3). Table 3 also lists genes whose altered expression in skeletal muscle post 2-AA treatment could lead to metabolic dysfunction, including down-regulation of the PGC-1β, PPAR-γ lipid metabolism genes, the UCP2 mitochondrial uncoupling: proton leackage gene, and Sirt1, which has been proposed to lie at the center of a loop regulating the actions of PGC and PPARs [50]; and up-regulation of the UCP3 mitochondrial uncoupling: proton leackage gene. Furthermore, the protein expression level of PGC-1β, PPAR-γ and Sirt1 was significantly dampened in mouse skeletal muscle cell line C2C12 (Fig. 4) following 2-AA treatment, corroborating with our mouse transcriptome data. This dampening effect was not due to 2-AA cytotoxicity (Fig. 5).

**Table 2:** ^1^H NMR HRMAS analysis on gastrocnemius muscle from 2-AA treated and control mice.

Chemical Shift PPM	Chemical group	Control	4 days Post 2-AA	Percent Change	p-value
1.30	(C**H**_2_)_n_	0.14±0.01^a^	0.08±0.02	+118.9	0.029^b^

**Footnote:** ^a^ values (μmol/g muscle) are means ± standard errors from 8 samples per group; ^b^ p value for comparisons between 2-AA treated and control mice obtained with the Student's t-test; + indicates increase

**Table 3:** Differential expression of key metabolic genes in skeletal muscle from 2-AA treated mice versus untreated control mice.

Gene Bank Accession No.	Name of Gene	Gene Symbol	GO Biological Process	Fold Change	p-value
BB345784	Insulin receptor substrate 1	Irs1	Insulin signaling	−6.225	0.0382
NM_010514	insulin-like growth factor 2	Igf2	Insulin signaling	−2.23	0.0309
BG092290	insulin-like growth factor 2 receptor	lgf2r	Insulin signaling	−3.119	0.0106
NM_008344	insulin-like growth factor binding protein 6	lgfbp6	Insulin signaling	−3.832	0.0137
BF225802	insulin-like growth factor binding protein 5	Igfbp5	Insulin signaling	−3.208	0.0372
BB787243	insulin-like growth factor binding protein 4	Igfbp4	Insulin signaling	−8.463	0.0076
AV257512	insulin induced gene 2	Insig2	Insulin signaling	−2.768	0.0212
AB008453	solute carrier family 2 (facilitated glucose transporter), member 4	Slc2a4 or GLUT4	Glucose transport	−4.399	0.0438
NM_133249	peroxisome proliferative activated receptor, gamma, coactivator 1 beta	Ppargc1b (PGC-1β)	Lipid metabolism	−2.904	0.00823
NM_011146	peroxisome proliferator activated receptor gamma	Pparg (PPAR-γ)	Lipid metabolism	−18.38	0.00256
AI645527	uncoupling protein 3 (mitochondrial, proton carrier)	Ucp3	Mitochondrial uncoupling; proton leackage	+5.291	0.00213
AW108044	uncoupling protein 2 (mitochondrial, proton carrier)	Ucp2	Mitochondrial uncoupling; proton leackage	−2.3	0.00274
NM_019812	sirtuin 1 ((silent mating type information regulation 2, homolog) 1 (S. cerevisiae)	Sirt1	muscle development	−2.3	0.043

Values are the relative expression intensity of the 2-AA treated versus control mice after 4 d Gene annotations for biological functions are from the Gene Ontology Consortium and the Ingenuity database.

(+) Upregulation of genes compared with control untreated muscle.

(−) Downregulation of genes compared with control untreated muscle.

Table 4 and Figure 2B show that 2-AA also alters fatty acid oxidation. Several genes involved in lipid metabolism were down-regulated (Fig. 2B). Eleven fatty acid oxidation genes were differentially expressed post 2-AA treatment, with most being down-regulated (Table 4). These genes encode proteins that increase the non-esterified fatty acids cytosolic pool, and function in fatty acid β-oxidation. Their reduced expression could contribute to muscle lipid accumulation and the lipid metabolism dysfunction produced by 2-AA. Plasma FFAs were not altered by 2-AA treatment.

**Table 4:** Differential expression of genes involved in fatty acid oxidation in mouse skeletal muscle at 4-AA treatment versus control muscle.

Gene Bank Accession No.	Gene Name	Fold Change	p- value
BG060909	stearoyl-Coenzyme A desaturase 2	+10.69	0.00507
NM_009127	stearoyl-Coenzyme A desaturase 1	−4.379	0.0249
NM_010726	phytanoyl-CoA hydroxylase	−4.708	0.000639
NM_010726	phytanoyl-CoA hydroxylase	−4.708	0.000639
AK017272	lipoprotein lipase	−8.606	0.00755
AK017272	lipoprotein lipase	−8.606	0.00755
AK017272	lipoprotein lipase	−8.606	0.00755
AK017272	lipoprotein lipase	−8.606	0.00755
BB114220	L-3-hydroxyacyl-Coenzyme A dehydrogenase, short chain	−6.56	0.002
AV018774	L-3-hydroxyacyl-Coenzyme A dehydrogenase, short chain	−5.24	0.00184
NM_008212	L-3-hydroxyacyl-Coenzyme A dehydrogenase, short chain	−5.945	0.0167
NM_010023	dodecenoyl-Coenzyme A delta isomerase (3,2 trans-enoyl-Coenyme A isomerase)	−5.248	0.00263
AK002555	acetyl-Coenzyme A acyltransferase 2 (mitochondrial 3-oxoacyl-Coenzyme A thiolase)	−4.842	0.0126
AK002555	acetyl-Coenzyme A acyltransferase 2 (mitochondrial 3-oxoacyl-Coenzyme A thiolase)	−7.626	0.00187
NM_133249	peroxisome proliferative activated receptor, gamma, coactivator 1 beta	−2.904	0.00823

(+) Upregulation of genes compared with control untreated muscle.

(−) Downregulation of genes compared with control untreated muscle.

In addition to this, the transcriptome data shows that stress activated protein kinase (SAPK) pathway genes are significantly downregulated (Table 5) in mouse skeletal muscle at 4 days 2-AA treatment. Therefore, it confirms the specific action of 2-AA in skeletal muscle.

**Table 5:** Downregulation of stress activated protein kinase (SAPK) pathway genes in mouse skeletal muscle at 4 days 2-AA treatment versus control muscle.

Gene Bank Accession No.	Gene Name	Fold Change	p-value
BM119623	activating transcription factor 2	−2.160	0.0335
BG071068	guanine nucleotide binding protein (G protein), beta polypeptide 1	−2.770	0.0165
AV021455	guanine nucleotide binding protein (G protein), gamma 2	−4.480	0.0199
BQ175363	mitogen-activated protein kinase kinase kinase 9	−8.400	0.00541
NM_009582	mitogen-activated protein kinase kinase kinase 12	−2.012	0.0455
AF220195	mitogen-activated protein kinase 8 interacting protein 2	−62.110	0.0116
AF262046	mitogen-activated protein kinase 8 interacting protein 3	−3.759	0.0119

(−) Downregulation of genes compared with control untreated muscle.

### 2-AA affects skeletal muscle function

**Figure 6:**
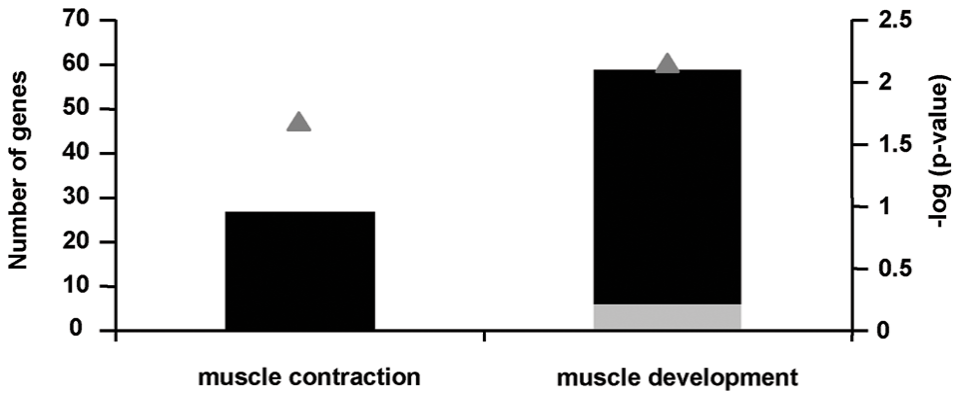
2-AA treatment down-regulates muscle function. Black bars indicate the number of down-regulated genes; gray bars indicate the number of up-regulated genes, in the skeletal muscle of mice 4 days post 2-AA treatment versus control mice (left vertical axis). The negative log10 of p-values represented by gray triangles are indicated on the right vertical axis.

Since 2-AA reduces ATP synthesis rate and energy production gene expression in skeletal muscle while reprograms the expression of key metabolic genes as presented above, we carried out further analysis and physiological studies to directly examine whether the 2-AA effects on metabolic gene expression and ATP synthesis impact muscle function. Our results show that the expression of all muscle contraction-related genes is significantly down regulated (Table 6 and Fig. 6) as well as muscle development–related genes (Fig. 6) indicating thus muscle dysfunction 4 days after 2AA treatment. In addition, Table 7 shows that at 4 days post 2-AA treatment, the absolute twitch tension of tibialis muscle was significantly reduced in the 2-AA versus the control mice. The evoked first single twitch tensions of tibialis muscle during TOF stimulation in the 2-AA mice were reduced to 72% on the left side and 80% on the right side, compared to the control mice (p<0.05). The maximum response of tetanic stimulation (Tmax) also was significantly different on the left side of 2-AA mice decreased 75% versus the controls.

**Table 6:** Downregulation of muscle contraction genes in mouse skeletal muscle at 4-AA treatment versus control muscle.

Gene Bank Accession No.	Gene Name	Fold Change	p-value
AK003186	tropomyosin 2, beta	−2.822	0.0291
NM_010867	myomesin 1	−6.41	0.0203
BM246564	phosphodiesterase 4B, cAMP specific	−2.161	0.0488
AK002271	tropomyosin 1, alpha	−3.175	0.021
BI248947	caldesmon 1	−5.736	0.0334
BC003284	WD repeat domain 21	−3	0.0288
BC024358	tropomyosin 2, beta	−3.439	0.0359
AY094172	calcium channel, voltage-dependent, beta 1 subunit	−2.317	0.0211
AA245637	ATPase, Ca++ transporting, cardiac muscle, slow twitch 2	−2.121	0.0402
X53753	tropomyosin 3, gamma	−2.399	0.0426
BC025840	titin	−2.281	0.0397
AK013026	annexin A6	−2.847	0.00242
AK010153	titin	−3.873	0.0149
AW558570	endothelin receptor type A	−4.527	0.0147
BB288010	myomesin 2	−14.32	0.0221
BB705075	Calponin 3, acidic (Cnn3), mRNA	−2.413	0.0044
BM122177	titin	−2.901	0.0328
BB478751	titin	−3.778	0.0137
NM_033268	actinin alpha 2	−4.234	0.0349
BC026142	myosin, heavy polypeptide 11, smooth muscle	−3.538	0.00149
NM_022314	tropomyosin 3, gamma	−2.607	0.0251
BB474208	myomesin 2	−8.349	0.039
BI663014	calponin 2	−2.342	0.00402
BB833102	calponin 3, acidic	−3.216	0.0354
AW108242	RIKEN cDNA 8030451F13 gene	−2.775	0.0286
BM232388	tropomyosin 1, alpha	−4.137	0.00079
AV241307	myomesin 2	−10.55	0.0258

(−) Downregulation of genes compared with control untreated muscle.

**Table 7:** Absolute wet-weights of tibialis (TA), soleus (So) and gastrocnemius (GC) muscles, and absolute twitch tensions (ST; single twitch, T_max_; maximum response of tetanic stimulation at 50 Hz for 5 sec) of tibialis muscle, at 4 days post 2-AA treatment, versus corresponding control muscle.

Wet weight (mg) and twitch height (g)	Control		2-AA	
	Left	Right	Left	Right
TA	54.0±3.2	54.7±2.4	45.7±3.5	47.3±2.2
So	8.1±0.8	8.0±0.5	7.8±0.3	8.4±0.8
GC	153.0±8.3	152.7±6.4	141.3±8.1	143.0±8.0
ST	**44.6±1.3** §	**45.0±1.7** ¶	**32.2±3.4** §	**35.8±1.9** ¶
T_max_	**121.0±7.0** †	118.7±5.0	**90.9±7.0** †	98.5±4.3
*N*	6		6	

Values are means ± SEM. Differences between the two sides of the 2-AA group and control group were compared using one-way ANOVA test and Bonferroni's multiple comparison as a *Post hoc* testing. Differences were assumed to be significant if the p-value was <0.05. There are no significant differences in wet muscle weights between the experimental and control mice (p>0.05). The twitch heights of single twitch stimulation and tetanic stimulation show significant differences between control and 2-AA mice (p<0.05).

§ Significant difference of single twitch height between control and 2-AA group in left side (p = 0.043).

¶ Significant difference of single twitch height between control and 2-AA group in right side (p = 0.047).

† Significant difference of maximum response of titanic stimulation between control and 2-AA group in left side (p = 0.011).

Lt: Left side of muscle.

Rt: Right side of muscle.

## Discussion

We show here that 2-AA, a diagnostically important bacterial interkingdom infochemical, leads to skeletal muscle dysfunction, and induces a biological signature of mitochondrial dysregulation and insulin resistance. Our results demonstrate that 2-AA down-regulates genes, including PPAR-γ, PGC-1β and Sirt1 that function in mitochondrial biogenesis, oxidative phosphorylation, and metabolic pathways, including insulin signaling, glucose transport, energy production, fatty acid oxidation, and skeletal muscle function. PPARs, in conjunction with PGC-1 and Sirt1, activate oxidative metabolism genes, and mediate insulin sensitivity in skeletal muscle; and their dysregulation could underlie metabolic dysfunction [50]. Such down-regulation may be mediated via an upstream regulatory pathway involving mitochondrial uncoupling, and ultimately lead to the skeletal muscle dysfunction observed here, and in burned skeletal muscle [35], [36], [51]. To our knowledge, 2-AA is the first QS molecule seen to promote these effects.

The 2-AA mediated suppression of PPAR-γ correlates with the reduced rate of ATP synthesis in the skeletal muscle of 2-AA treated mice (Fig. 1 and Table 1), as well as the downregulation of glucose transporter and insulin signaling genes (Table 3). The transcription factor PPAR-γ plays a pivotal role in maintaining oxidative phosphorylation, insulin-mediated signaling, and glucose homeostasis, along with other co-activators like PGC-1 and Sirt-1 [50], [52]–[54]. As such, our results suggest that PPAR-γ suppression could affect energy homeostasis and insulin-induced glucose metabolism in skeletal muscle, which could then cause mitochondrial dysfunction and insulin resistance [55], [56]. Indeed, PPAR-γ downregulation in CF deficient cells leads to chronic inflammation [24], [57]. Also, PPAR-γ overexpression can rescue mitochondrial dysfunction in chronic mitochondrial disease [58].

That 2-AA down-regulates PGC-1β and upregulates UCP3 (Table 3) is consistent with its reduction of ATP synthesis rate and down-regulation of PPAR-γ. The PGC-1β protein plays a major role in mitochondrial metabolism, as it increases mitochondrial biogenesis and muscle cell respiration [8]; and is hypothesized to have a central role in regulating energy homeostasis and metabolism [23], [35], [36]. As such, PGC-1β down-regulation could lead to the down-regulation of oxidative phosphorylation genes (OXPHOS) [35], [36], as their expression is down-regulated by 2-AA (Fig. 2A). Consequently, these changes could lead to mitochondrial uncoupling, as suggested by the up-regulation of UCP3 (Table 3). To this end, our data suggest that 2-AA dysregulates components of mitochondrial metabolism at the transcriptional level, to result in skeletal muscle dysfunction (Table 6–7). Interestingly, PPAR-γ was suggested to act as the N-acyl-homoserine lactone (AHL) signaling molecule N-3-oxododecanoyl homoserine lactone (3-oxo-C_12_-HSL) mammalian receptor and it is shown to function as an antagonist of PPAR-γ transcriptional activity and inhibit the DNA binding ability of PPAR-γ [59].

**Figure 7:**
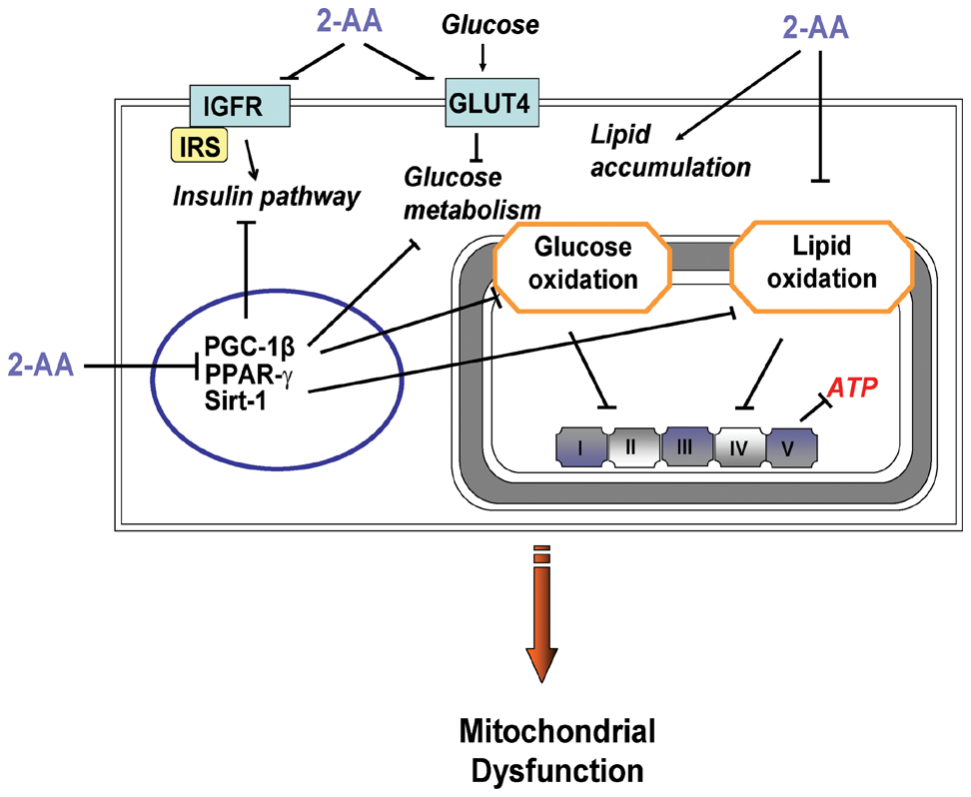
Schematic model for 2-AA mediated molecular mechanisms of mitochondrial dysfunction in skeletal muscle. 2-AA triggers mitochondrial dysfunction via the down-regulation of peroxisome proliferative activated receptor, gamma, coactivator (PGC)-1β, peroxisome proliferator activated receptor (PPAR)-γ, sirtuin (Sirt)-1, the insulin signaling pathway, overall energy metabolism, and increased lipid accumulation.

Cells overexpressing PGC-1β exhibit increased activity of ATP consuming reactions [8]. Here downreguation of PGC-1β coincides with reduced ATP synthesis rate. That 2-AA also reduces IRS1 expression (Table 3) suggests that PGC-1β, which is downregulated here, perturbs IRS1 expression, and consequently effects insulin resistance in skeletal muscle. As such, PGC-1 may contribute to insulin resistance to then mediate inflammation and disrupt glucose homeostasis [13]. In addition, the 2-AA mediated down-regulation of Sirt1 (Table 3) is possibly associated with energy expenditure and insulin sensitivity, and likely reflects the impaired mitochondrial function [60]. The pleotropic transcription factor PGC-1 functions in the regulation of differential gene expression, in conjunction with other transcription factors (e.g., PPAR, SIRT-1), in cells exhibiting high energy-demands, including skeletal muscle [8], [60], [61]. Here, 2-AA down-regulates PGC-1β, IRS1, IGFs and GLUT4 expression (Table 3), indicating a connection between PGC-1β and metabolic genes. This suggests that PGC-1β acts to regulate energy metabolism genes in skeletal muscle, and that mitochondrial dysfunction leads to the down-regulation of the insulin signaling pathway, and impaired systemic insulin activity (Fig. 7). To this end, our findings suggest a more general role for PGC-1β in skeletal muscle metabolism, and possibly in the progression of chronic infection [62], in addition to the insulin resistance seen in obese and/or type 2 diabetic patients [48], [49], [63], and CF patients with a high incidence of diabetes [25].

2-AA also downregulates genes involved in fatty acid oxidation, energy production, intermediary metabolism, and the TCA cycle (Table 4; Fig. 2b) This downregulation could in turn lead to a demand for increased muscle contractile gene expression to fulfill functional requirements; however, our results show that 2-AA reduces the expression of these genes (Fig. 6). This reduction could underlie the observed muscle function impairment (Table 7), and correspond to the impaired muscle strength in CF patients [64]. Our data suggest that this functional impairment may be due to reduced mitochondrial coupling, as both ATP synthesis and TCA flux are reduced, and the ratio of ATP synthesis and TCA flux can serve as an index of mitochondrial coupling [37].

*In vivo* ^31^P magnetization-transfer can non-invasively determine the unidirectional flux of P_i_ to ATP (“ATP synthesis”), and has been used to show that abnormal mitochondrial function occurs in obesity and diabetes [37]. This technique [65], [66] has enabled us to noninvasively measure fast enzyme reaction exchange rates, to provide an index of the net skeletal muscle rate of oxidative ATP synthesis catalyzed by mitochondrial ATPase. This is by definition proportional to the oxygen consumption rate by the P/O ratio, e.g., the ratio of the net rate of ATP synthesis by oxidative phosphorylation to the rate of oxygen consumption [67],[68]. Unidirectional ATP synthesis flux, measured by NMR, is thought to primarily reflect flux through F_1_F_0_-ATP synthase, with negligible influence of the coupled glyceraldehyde-3-phosphate dehydrogenase (G3PDH), or phosphoglycerate kinase (PGK) reactions [66]. Although the net glycolytic contribution of G3PDH and PGK to ATP production is small, versus oxidative phosphorylation, these enzymes occur at near equilibrium, allowing high unidirectional ATP production. As such, we assume the contribution of glycolytic reactions to unidirectional ATP synthesis flux is negligible.

IMCLs levels are increased in mouse gastrocnemius muscle following 2-AA treatment, as assessed by HRMAS ^1^H NMR spectroscopy (Table 2 and Fig 3). Although the source of these accumulated lipids is beyond this study, it has been shown that EMCLs, IMCLs, and triglycerides all contribute to cellular lipid peaks [42], [69], [70]. IMCL probably serve as energy substrates for oxidative metabolism [71], and can be mobilized and utilized with turnover rates of several hours [72]. Furthermore, the lipid peak at 1.4 ppm in Figure 3 is attributed to methylene protons of intra-myocellular triglyceride acyl chains, primarily due to IMCL [42], to suggest that the increase in NMR-visible lipids at 1.4 ppm post 2-AA treatment is primarily due to increased IMCL. This is further supported by human studies [73],[74] where IMCLs were suggested to serve as metabolic biomarkers of insulin resistance. Nevertheless, lipid accumulation may reflect increased inflammation versus mitochondrial dysfunction [13], although 2-AA immunomodulation is characterized by decreased inflammation [31]. Here, increased IMCLs were associated with altered expression of key regulators of insulin signaling and glucose metabolism (Table 3), which can lead to insulin resistance in association with mitochondrial dysfunction. To this end, 2-AA might mediate insulin resistance, as IMCLs are biomarkers of such resistance, and it down-regulates IRS1, IGFs, and GLUT4. Nevertheless we have not directly demonstrated insulin resistance by traditional methods.

Although the exact mechanism of abnormal insulin function is unknown, there is increasing evidence that plasma FFAs may act to induce insulin resistance [75]. However, intramyocellular accumulation of toxic FFA metabolites, *i.e*. fatty acyl-coenzyme A, diacylglycerol, and ceramides, has been also shown to impair insulin signal transduction, glucose transport/phosphorylation, and glycogen synthesis [46], [75], [76]. Because fatty acid metabolism and glucose levels are closely linked, we propose that the observed accumulation of lipids or triglycerides in muscle, may lead to insulin resistance [77], although we did not observe any alteration in plasma FFA, in agreement with the unchanged FFA levels in CF patients [25]. In addition, previous studies have suggested that muscle activity of lipoprotein lipase (LPL) is related to insulin resistance [78] and that insulin may downregulate the activity of lipoprotein lipase in skeletal muscle [79]. This is in accordance with the decreased expression of the LPL gene in the skeletal muscle of the 2-AA treated mice (Table 4).

CF patients often develop skeletal muscle wasting [80], and insulin resistance [25], [81]. 2-AA mediated downregulation of muscle gene functions is associated with tissue loss and low ATP synthesis rate in skeletal muscle. Our results suggest that 2-AA may act as an important contributor of host metabolic alterations in disease states characterized by chronic infection. CF is one such syndrome, where the associated pathophysiology stems as much from the underlying gene mutation as from the colonization and adaptation of particular flora in the mucosal pulmonary milieu [82]. In acute infections, metabolic alterations are short-lived and reverse quickly [83], [84], whereas in chronic infections the protracted metabolic response leads to muscle wasting [85], [86]. *P. aeruginosa* and 2-AA in chronically infected CF lungs is considered pathognomonic [32], [33], [87], [88]. As such, further investigation into the potential clinical significance of 2-AA is warranted, 2-AA modulates immune responses to promote host tolerance and chronic infection [31]. To this end, it is possible that the 2-AA mediated changes of the host metabolome may further contribute to host tolerance and chronic *P. aeruginosa* infection. Indeed, inhibition of PPAR-γ reduces the phagocytic activity of macrophages [89], which may serve to evade the host defense mechanism, and possibly favor infection and allow long term bacterial presence promoted by 2-AA [31]. Our results are consistent with other chronic infections, including HIV [90], tuberculosis [91], chronic *Escherichia coli* in skeletal muscle [92], and chronically infected CF patients [93].

## Conclusion

Based on our multidisciplinary results, Figure 7 proposes a novel mode of action for the bacterial pathogen infochemical, 2-AA, to mediate host metabolic dysregulation that results in mitochondrial dysfunction, and potentially insulin resistance, in skeletal muscle. The metabolic changes promoted by 2-AA may be clinically relevant in molecular medicine in general, and CF in particular, as: a) ATP synthesis rate and IMCLs can be measured clinically using non-invasive and non-irradiating metabolomic assays; b) PGC-1β activity may be induced; and c) PGC-1β agonists may alleviate insulin resistance and prevent damage in organs remote from the infection site. In addition, our results provide further insights into the molecular and metabolic processes mediated by 2-AA that accompany infections caused by 2-AA producing pathogens, including *Pseudomonas aeruginosa* and *Burkholderia* species [32], [33], as well as 2-AA-related molecules produced by other pathogens.
